# Intuition-guided Reinforcement Learning for Soft Tissue Manipulation with Unknown Constraints

**DOI:** 10.34133/cbsystems.0114

**Published:** 2025-04-14

**Authors:** Xian He, Shuai Zhang, Jian Chu, Tongyu Jia, Lantao Yu, Bo Ouyang

**Affiliations:** ^1^ School of Management, Hefei University of Technology, Hefei, China.; ^2^ Key Laboratory of Process Optimization and Intelligent Decision-Making (Ministry of Education), Hefei University of Technology, Hefei, China.; ^3^ Faculty of Urology, Third Medical Center, Chinese PLA General Hospital, Beijing, China.; ^4^ Independent Researcher, San Jose, CA, USA.

## Abstract

Intraoperative soft tissue manipulation is a critical challenge in autonomous robotic surgery. Furthermore, the intricate *in vivo* environment surrounding the target soft tissues poses additional hindrances to autonomous robotic decision-making. Previous studies assumed the grasping point was known and the target deformation could be achieved. The constraints were assumed to be constant during the operation, and there were no obstacles around the soft tissue. To address these problems, an intuition-guided deep reinforcement learning framework based on soft actor-critic (ID-SAC) was proposed for soft tissue manipulation under unknown constraints. The SAC algorithm is automatically activated upon encountering an obstacle, and the designed intuitive manipulation (IM) strategy is used to pull soft tissues toward the target shape directly when the obstacle is distant. A regulator factor is designed as an action within this framework to coordinate the IM approach and the SAC network. A reward function is designed to balance the exploration and exploitation of large deformations. Simultaneously, we proposed an autonomous grasp point selection neural network to prevent the impractical selection of grasp points, ensuring they can reach the target while avoiding grasping lesions and constrained areas. Successful simulation results confirmed that the proposed framework can manipulate the soft tissue while avoiding obstacles and adding new positional constraints. Compared with the SAC algorithm, the proposed framework can markedly increase the robotic soft tissue manipulation ability by automatically adjusting the regulator factors.

## Introduction

Robot-assisted minimally invasive surgery promises to improve the control accuracy of instruments through tremor cancellation and amplification ratio of the master-slave system. Over the past two decades, many surgeons have performed robot-assisted laparoscopic surgeries, such as radical laparoscopic prostatectomy assisted by the da Vinci surgical system. However, the autonomy of surgical robots in soft tissue manipulation remains limited, with most surgeons still manually controlling instruments via the teleoperation system. Roboticists have focused on increasing the level of autonomy of surgical robots [1], including automatic suturing [2,3] and cutting [4]. A key issue in autonomous robotic surgery is the automatic soft tissue manipulation, which is often performed before suturing and cutting.

Autonomous soft tissue manipulation with variable constraints is a challenging problem. Although previous studies have successfully manipulated cloth and string-like objects to the desired deformation, only a few studies have been conducted on soft tissue manipulation [5–8]. Unlike clothing and string-like deformable objects, soft tissue is constrained by other connected organs and instruments in an *in vivo* environment. These connections may be changed by pulling and cutting operations. Moreover, the surrounding tissues or instruments should be avoided during soft tissue manipulation.

The dynamics of deformable objects manipulated by instruments have been explored in preliminary studies. The mass-spring model is often used to simulate deformable objects [9]. However, it is inaccurate for large deformations of soft tissues because it does not inherently capture the continuous material properties and nonlinear behavior of soft tissues under stress and strain. The finite-element model is another effective approach for simulating soft tissues. However, this model is sensitive to soft tissue constraints and external disturbances caused by mesh quality and density, material properties, boundary conditions, and constraints [10]. Few studies have also explored active deformation control under variable and unknown connection constraints of the soft tissue, which is a common situation in laparoscopic surgery.

Moreover, soft tissues have infinite degrees of freedom, which poses challenges in estimating their shape and physical parameters *in vivo*. Furthermore, a carefully designed controller based on dynamics may become unstable owing to uncertainties and inaccurate estimation parameters [11]. To design a controller, nonlinear systems are often simplified or linearized around a set of operating points. Although this simplification facilitates the design process, it can introduce errors. Additionally, measurement noise and disturbances can further reduce the quality of the information available for control. Therefore, achieving deformation control strategies based on an accurate model in real robotic surgeries is challenging.

Conversely, some researchers have investigated model-less approaches for soft tissue manipulation. For instance, Navarro-Alarcon et al. [5,12] attempted to estimate the deformation Jacobian matrix of soft tissues in real time and designed an adaptive controller with visual feedback. The deformation Jacobian matrix is a linear approximation of the deformation model in a short time, which works efficiently when the instruments move slowly. Hu et al. [13] suggested using a deep neural network (DNN) to approximate the map between the instrument’s movement and the deformation. An online learning approach was used to update the neural network. The learned DNN controller may fail because of the limited online data when an external force is suddenly applied to the soft tissue.

Recent success in reinforcement learning, such as solving the Rubik’s Cube with a robotic hand [14], is promising for soft tissue manipulation. Pedram et al. [15] used Q-learning to manipulate soft tissues, in which the agent had only 25 possible actions. Shahkoo and Abin [6] compared reinforcement and imitation learning approaches for soft tissue manipulation. Both approaches can perform manipulation tasks, but imitation learning can limit exploration. In some deep reinforcement learning frameworks, the policy network is initialized through imitation learning to accelerate the training process [16,17]. However, demonstrating all soft tissue manipulation scenes is prohibitive due to the high-dimensional deformation space and variable contact constraints. The distribution between the demonstrations and test scenes may be non-identical as well. Therefore, an approach that can guide the policy network throughout the entire training process is required.

To simplify the problem, many existing algorithms assume that the manipulation point is appropriately selected before the deformation manipulation and that the soft tissue can be manipulated to achieve the target deformation. However, the grasping point should be reselected to avoid over-deformation during the training process, particularly when unknown constraints are introduced into the tissue. In recent years, some researchers have explored the manipulation point regulator issues. Viswanath et al. [18] presented a grasping point selection method by defining a disentangling hierarchy over cable crossings for the robotic untangling of cable. Huang et al. [19] proposed an active regulator algorithm for soft tissue manipulation with nonfixed contact, where the contact area can be adjusted by sliding the end effector, although the grasping point remains fixed unless the grasper is opened. Therefore, grasping point selection for soft tissue manipulation remains an unresolved problem.

We proposed an intuition-guided deep reinforcement learning framework for soft tissue manipulation under unknown constraints. The agent is similar to the brain, which has two modes of thinking: intuitive and deliberate. The intuitive mode is characterized by a simple manipulation approach in which the agent consistently pulls the soft tissue toward the target deformation. The SAC algorithm [20,21] is applied to tackle complex manipulation issues, such as avoiding obstacles. The agent coordinates the two modes by regulating a weight, which is treated as an action and updated by the manipulation policy based on the previous state. Grasping point selection is treated as a contextual bandit problem [22]. The agent learns the policy for grasping point selection using deep Q-learning [23]. The final state-action value, which represents the success rate of each grasping point, guides the agent in selecting the optimal grasping point to achieve the desired deformation.

In this study, we explored point-, curve-, and region-based deformation tasks. A complex environmental model must be established for curve- and region-based deformation tasks. This includes modeling the dynamic properties of soft tissues, the complexity of anatomy, and uncertainties in surgical scenarios. Deep reinforcement learning has proven capable of learning models directly from environmental interactions. The feature point farthest from the target determines the reward in curve- and region-based tasks. To overcome these long horizons, a new reward function was applied to large deformation tasks. Using piecewise reward functions in reinforcement learning for soft tissue manipulation, particularly with large deformations, can help the learning algorithm recognize and reinforce successful strategies at different stages of the manipulation task. It also allows for finer manipulation of the learning objectives, making it easier to guide the algorithm toward the desired outcomes in complex situations. The experimental results confirm that the proposed framework can select an appropriate grasping point and successfully manipulate deformation. The deliberate mode is activated when obstacles appear around the tissue or when external forces are applied. Furthermore, the proposed framework can potentially accelerate training procedures and improve generalization.

The main contributions of this study are summarized as follows:

1. We proposed an intuition-guided deep reinforcement learning framework based on soft actor-critic (ID-SAC) for manipulating soft tissue deformation under unknown constraints, unlike previous studies that assumed that soft tissue constraints are constant and have no obstacles. The proposed framework does not initialize the policy with demonstrations; rather, it incorporates an IM strategy and deliberates the policy through the action of the agent.

2. We presented an autonomous grasping point selection algorithm using a soft tissue manipulation framework. The state-action value of the proposed algorithm can be used to determine the optimal grasping point, given the target deformation.

3. We explored three types of deformation manipulation tasks using reinforcement learning. A uniform reward function was designed for these tasks, which can guide exploration and exploitation in a large workspace.

### Overview and Problem Statement

**Fig. 1.**
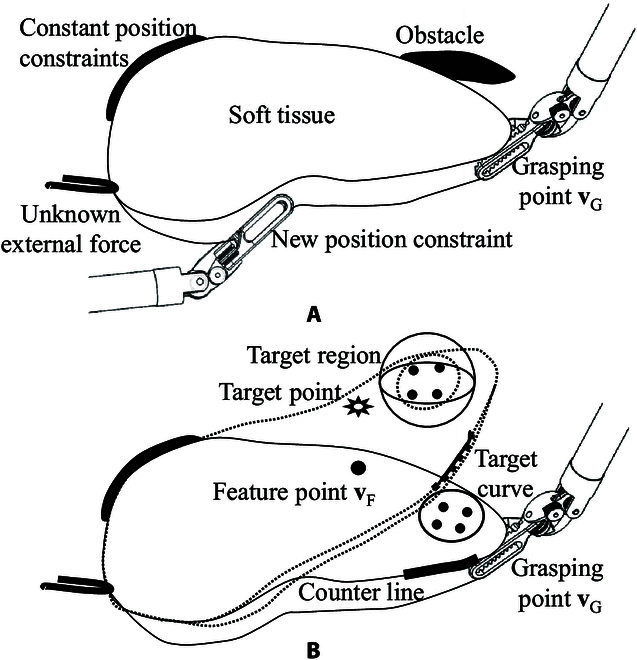
Conceptual representation of soft tissue manipulation. (A) Constraints of soft tissue manipulation. (B) Three types of soft tissue deformation.

Active soft tissue manipulation was primarily developed to assist surgeons in this study. The agent functioned similarly to a physician assistant during robotic surgery. The deformation of soft tissue *X* is described as the set of feature points $x_{i}\left(t\right)$ on the surface, which helps identify constraints. Although a fast point feature histogram (FPFH) [24] can encode deformation, it is not intuitive for surgeons. Soft tissue deformation is subject to fasciae and instruments in an *in vivo* environment. Position constraints are defined as tissue parts that cannot move during manipulation, denoted as $x_{p}^{c}\left(t\right)=x_{p}^{c}\left(0\right)$, where $x_{p}^{c}\in X$, p=1,…,P, and *P* represent the total number of constant position constraints. Two types of unknown soft tissue constraints are considered (Fig. 1A): new position constraints $x_{q}^{a}\left(t\right)=x_{q}^{a}\left(\tau \right)$ and unknown force constraints $f_{l}\left(t\right)$, where $x_{q}^{a}\in X$ and τ are a piecewise function concerning time *t*, q=1,…,Q, l=1,…,L, and *Q* and *L* are the total number of each constraint, respectively.

Deformation manipulation tasks mainly include pulling a tissue part to the target region or a needle tip and shaping the contour line to the desired curve. Surgeons rarely cause global deformation of soft tissue during surgery. Therefore, we presented three definitions of local target deformations for soft tissue manipulation with visual feedback (Fig. 1B).

1. Position-based deformation: The target deformation is determined by the position $v^{d}$ of a feature point $v^{f}\in X$. Position-based deformation manipulation is a basic operation task in soft tissue manipulation.

2. Curve-based deformation: The target deformation is described by a curve. This paper determined the target curve by discrete points $y_{j}$. The relative position of discrete points before and after manipulation remains unchanged.

3. Region-based deformation: A tissue part is manipulated to the target region for cutting or exposing tissue. The region is determined by the center $o_{h}$ of a circle and its diameter $d_{c}$. This paper determined the target regions by discrete points $y_{j}$.

Most robotic systems for laparoscopic surgery only provide stereo vision; however, the surgeon can successfully perform the surgery. It is also challenging to detect forces from the environment during robotic surgery. Therefore, we explored soft tissue manipulation by manipulating the grasper position $v^{g}\left(t\right)$ under unknown constraints. Moreover, the agent must avoid obstacles around tissues. Based on the above definitions, soft tissue manipulation under unknown constraints can be formulated as follows:

$$\begin{array}{c} \begin{array}{l} \underset{v^{g}\left(t\right)}{\text{arg}\text{min}}\;\mu \left(x_{1},\ldots ,x_{K},y_{1},\ldots ,y_{M}\right)\\ s.t.x_{p}^{c}\left(t\right)=x_{p}^{c}\left(0\right),p=1,\ldots ,P\\ \;x_{q}^{a}\left(t\right)=x_{q}^{a}\left(\tau \right),q=1,\ldots ,Q\\ \;x_{i}\left(t\right)=x_{i^{\prime }}\left(t\right)+\omega \left(f_{l}\left(t\right)\right),l=1,\ldots ,L\\ \;v^{g}\left(t\right)-o_{b}^{h}\notin B_{h}^{O},h=1,\ldots ,H\\ \;v^{g}\left(t\right)\in B^{s} \end{array} \end{array}$$ (1)

where $\mu \left(\cdot \right)$ denotes the measure function of the error between the current state and the target deformation, $y_{j}$ represents the discrete points for defining the target deformation, $x_{i}^{\prime }$ indicates the position of the *i*th feature point without disturbances, $B^{s}$ is the position bound, $o_{b}$ is the center of the *h*th obstacle $O_{h}$, $B_{h}^{o}$ denotes obstacle space, and $\omega \left(f_{l}\left(t\right)\right)$ represents the displacement caused by the *l*th unknown force constraint. Manipulation and planning algorithms based on models are unreliable in solving the problem because of unknown constraints and disturbances from the *in vivo* environment. Therefore, we explored model-free reinforcement learning to solve soft tissue manipulation, as it is independent of a predefined model of tissue behavior. This approach learns the optimal manipulation strategy directly from interacting with the environment. The agent learns to handle unknown constraints and disturbances through trial and error.

## Methods

### Framework overview

**Fig. 2.**
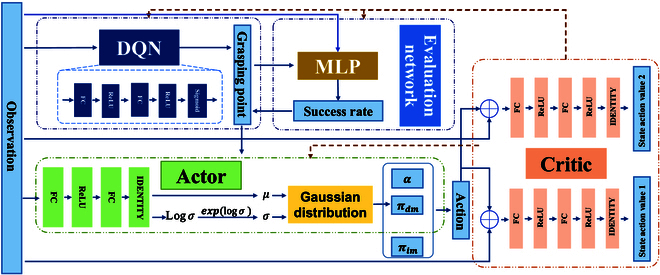
Conceptual representation of autonomous soft tissue manipulation network.

Although robots can perceive and manipulate objects precisely, their current autonomy remains limited. Moreover, the high-dimensional status space within the body and uncertain soft tissue deformations under variable constraints present several challenges for autonomous robotic surgery. Although doctors are unable to perform fine manipulations, their understanding of the surgical scene is substantial. Therefore, this study proposed a fusion strategy based on deep reinforcement learning that integrates the abilities of doctors and robots to address position-based soft tissue deformation manipulation tasks. This approach aims to enhance the intraoperative decision-making capabilities of robots, ultimately facilitating the improved execution of surgical tasks. All three proposed networks are multi-layer perceptron (MLP) with 2 hidden layers of 256 units (Fig. 2).

Step 1: Selection and evaluation of grasping points in autonomous soft tissue manipulation by robots. A deep Q-network-based algorithm was proposed to address the initial point selection issue in soft tissue manipulation. First, high-dimensional states were described and used as inputs to the network, which outputs the action value for each grasping point. The grasping point corresponding to the maximum action value was selected as the initial grasping point. Simultaneously, a grasp point quality assessment network was designed to predict the success rate of a selected grasp point for achieving the soft tissue manipulation task. The point selection operation was revised if the success rate during testing fell below a certain threshold. Subsequently, based on the selected grasp points, autonomous robotic manipulation uses a simulated IM mode akin to a physician’s approach to execute the manipulation task. Safety constraints were designed to ensure that when the soft tissue undergoes excessive deformation, the robot relinquishes the manipulation task. The network is updated through feedback from the manipulation trajectories of each point selection. A reward function was developed based on expert knowledge.

Step 2: Deep reinforcement learning-based fusion strategy for soft tissue manipulation modes. The SAC algorithm was used to execute soft tissue manipulation tasks after selecting initial grasping points in step 1, addressing the intricate and dynamic environment of soft tissue manipulation within the body. The regulatory factor is set as the action of the reinforcement learning algorithm to autonomously integrate multiple soft tissue manipulation modes, such that the manipulation strategy integrates the advantages of different manipulation modes and improves the robot’s ability to make autonomous decisions in complex scenes.


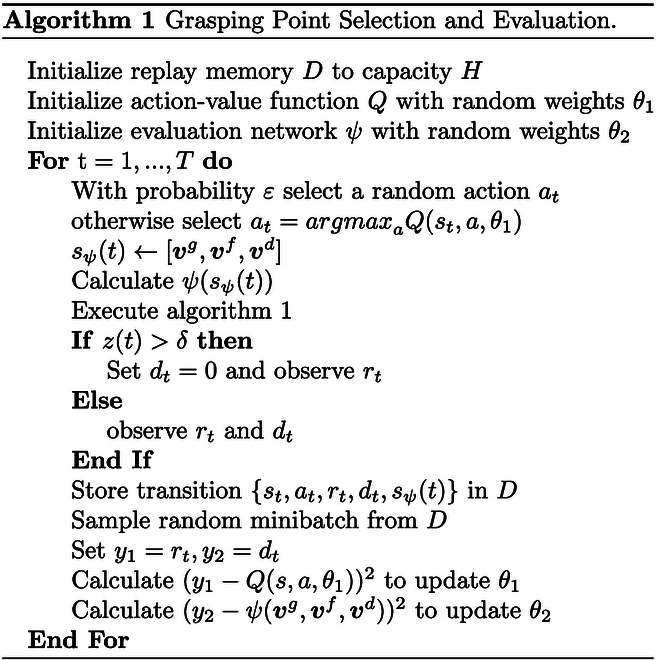


### Grasping point selection

The initial grasping point selection is a contextual bandit problem because the grasping point cannot be modified during soft tissue manipulation. The grasping point is similar to that of the slot machine. If the agent can manipulate the soft tissue to the target deformation, the reward is calculated based on the trajectory of the feedback points. However, the agent must determine when to abandon the manipulation task to prevent an over-deformation. We defined four indexes to evaluate the trajectory:

$$r_{d}=1-\sum ∥d\left(t\right)∥/\sum ∥d\left(0\right)∥$$ (2)

$$r_{g}=1-\beta_{g}\left(\sum ∥g\left(t\right)∥-\sum ∥g\left(0\right)∥\right.$$ (3)

$$r_{g0}=\beta_{g0}∥g\left(0\right)∥$$ (4)

$$r_{t}=1-\left(n-N^{\prime }\right)/N$$ (5)

where $d\left(t\right)=v^{d}-v^{f}$, $g\left(t\right)=v^{f}-v^{g}$, *n* is the total step, *N* is the maximum step, $N^{\prime }$ is a step threshold, $n=N^{\prime }$ if *n* is less than $N^{\prime }$, and $\beta_{g}$ and $\beta_{g0}$ are positive constants. The index $r_{d}$ means that the feature point $v^{f}$ is expected to be close to the target point $v^{d}$ during manipulation. If the majority of deformation errors exceed the initial error, the quality of the initial grasping point is considered poor. However, if it is close to the target deformation, the agent receives continuous rewards. Based on the positive feedback of the current grasping point, the larger this term indirectly indicates the shorter total distance of surgical control. The index $r_{g}$ evaluates the strain between the grasping point and the feedback points. If the index $r_{g}$ is small, it indicates that the agent may injure the soft tissue, even if the feedback points have reached the target. The index $r_{g0}$ constraints that the initial grasping point should not be far from the feedback points. The index $r_{t}$ evaluates the length of the manipulation episodes. After completing an episode, the agent receives a reward signal $R_{\mathrm{tr}}$:

$$R_{\mathrm{tr}}=1_{E}\rho_{\mathrm{tr}}R_{e}^{\text{in}}+\left(1-1_{E}\right)\rho_{\mathrm{tr}}R_{e}^{\text{out}}$$ (6)

where $\rho_{\mathrm{tr}}=\lambda_{t}r_{t}+\lambda_{d}r_{d}+\lambda_{g}r_{g}-\lambda_{g0}r_{g0}^{2}$; $\lambda_{\left(\cdot \right)}\in \left[0,1\right]$. $1_{E}$ is a symbol that takes a value of 0 or 1 based on the grasping point position. If the grasping point is at the edge of the soft tissue, $1_{E}=1$, the agent receives a reward of $\rho_{\mathrm{tr}}R_{e}^{\text{in}}$. If the grasping point is outside the edge, $1_{E}=0$, the reward is $\rho_{\mathrm{tr}}R_{e}^{\text{out}}$. This setting aligns better with the doctor’s operational understanding, such as tending to grab edge points and returning rewards for different grasp points based on the variable $1_{E}$. If the agent fails to manipulate the deformation, but the grasping point is located at the edge, the reward is set as $R_{\mathrm{se}}$. Otherwise, the reward is $R_{\mathrm{fe}}$. The total reward can be expressed as

$$R_{t}^{g}=\left(1-1_{A}\right)\left(1_{E}R_{\mathrm{se}}+\left(1-1_{E}\right)R_{\mathrm{fe}}\right)+1_{A}R_{\mathrm{tr}}$$ (7)

where when the feature point reaches the target point during manipulation to achieve the manipulation task, $1_{A}=1$; when it does not reach the target point and the manipulation fails, $1_{A}=0$.

During the training, a security constraint index $z_{s}$ is defined to abandon the manipulation task if the soft tissue is over-deformed:


$$z_{s}=\left\{\begin{array}{cc} 1, & \text{if}\;\sum_{i}^{i=N}1_{S^{\prime }}^{i}>\delta \\ 0, & \text{otherwise} \end{array}\right.$$ (8)

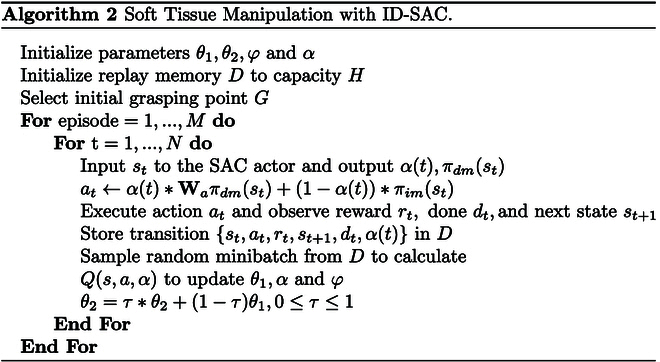

where $\begin{array}{c} S^{\prime }=\left\{s_{t}\;|\;\beta_{\mathrm{sc}}\left(|∥g\left(t\right)∥-∥g\left(0\right)∥|\right)>1\;\|\;∥d_{t-1}∥<\right. \end{array}$$\left.∥,d_{t-1},∥,<\right\}$ and δ is a threshold. $1_{S^{\prime }}^{i}$ represents the evaluation of the manipulation state at the *k*th moment of the manipulation process. If it is an unsafe manipulation state, $1_{S^{\prime }}^{i}=1$, and record it; otherwise, $1_{S^{\prime }}^{i}=0$. If the index $z_{s}$ exceeds the threshold δ, the agent abandons the deformation manipulation task.

To further ensure safety and expedite the process while enhancing efficiency, this study aimed to determine the success rate of the current grasp point selection process during manipulation. This information serves as a more intuitive reference for medical personnel in addition to setting safety constraints during manipulation. Therefore, this study designed a grasp point quality assessment network to predict the probability of achieving soft tissue manipulation tasks at the current grasp point. This network, structured based on a MLP, takes a triplet composed of the grasp point $v^{g}$ obtained from the deep Q-learning algorithm, the feature points $v^{f}$ on the soft tissue, and the target point $v^{d}$ as input. The network output is a success rate within the range of 0 to 1, whereas the ground truth is determined by the task completion value $d\left(t\right)$ achieved by the agent at that particular grasp point. During testing, the study identifies grasp points with a success rate below 0.8 as inadequate and initiates a reselection of the grasp points.

Deep Q-learning is used to train the grasping policy. The normalized final state-action value $Q_{\mathrm{sr}}$ indicates the success rate of each grasping point. The agent selects the grasping point based on the success rate Ω given the target deformation. The training details are presented in Algorithm 1.

### Reinforcement learning with manipulation knowledge

To deploy reinforcement learning in soft tissue manipulation, we must address two questions: what is the optimal initial grasping point given the target deformation, and how can the soft tissue be manipulated under unknown constraints? The first question depends on the second question in the reinforcement learning framework. If the agent is unable to manipulate the soft tissue effectively, the evaluation of the initial grasping point is considered inaccurate. To address this problem, we proposed a reinforcement learning framework with manipulation knowledge for soft tissue manipulation.

The state *S*(*t*) is the set of feedback points $x_{i}\left(t\right)$, target points $y_{j}$, grasping points $v^{g}\left(t\right)$, obstacles $o_{b}$, and the constant position constraint $x_{p}^{c}$. Inputting these features into the neural network assists the agent in identifying the unknown constraints during manipulation. The action $A_{t}\in {\mathbb{R}}^{3}$ is set as the grasper movement. Here, it is assumed that an appropriate initial grasping point is selected.

Similar to the brain, we designed two modes of thinking for the agent: intuitive and deliberate. We attempted to develop a simple manipulation approach $\pi_{\mathrm{im}}\left(S_{t}\right)$ from actual surgery. For instance, pulling a feature point toward a target point in the position-based deformation manipulation task, that is $\pi_{\mathrm{im}}\left(S_{t}\right)=K_{p}\left(v^{d}-v^{f}\right)$. $K_{p}$_,_ the proportional coefficient in the IM strategy is a constant and can be set based on experiments. Model-free fuzzy controllers are a good option. However, a simple manipulation approach can achieve only a few deformation tasks. The proportional control approach is limited in certain manipulation tasks, particularly for large soft tissue deformations because it relies on linear relationships between the input and output, which ignores the nonlinear properties of soft tissues. Deliberate mode should be activated in complex situations. The SAC is used to train the manipulation policy $\pi_{\mathrm{dm}}\left(S_{t}\right)$ in this mode. A key challenge is determining when to activate the deliberate mode, specifically, how to evaluate the reliability of each model. We set $\alpha \left(t\right)$ as an action of the actor. The output of $\pi_{\mathrm{dm}}\left(S_{t}\right)$ includes both the movement of the grasper and the complex index. To coordinate the two manipulation modes, the grasper movement is expressed as follows:

$$a_{t}=\alpha \left(t\right)*W_{a}\pi_{\mathrm{dm}}\left(S_{t}\right)+\left(1-\alpha \left(t\right)\right)*\pi_{\mathrm{im}}\left(S_{t}\right)$$ (9)

where $W_{a}=\left[I_{3}\;0\right]$ and $\alpha \left(t\right)\in \left[0,1\right]$. The reliability $\alpha \left(t\right)$ is updated in real time according to the state. When α is close to 0, the agent is inclined to use $\pi_{\mathrm{im}}$; however, when α is close to 1, the deliberate mode $\pi_{\mathrm{dm}}$ is used. The proposed framework can also be used to coordinate the behavior of the surgeon and the robot during robotic surgery.

We explored the reward design problem for the target deformations described by single or multiple points. The agent receives a penalty of $-\rho ∥d\left(t\right)∥$ at any time where $d\left(t\right)=v^{d}-v^{f}$, such that the agent tries its best to find the optimal trajectory. If the grasper moves out of the boundary, the agent receives a penalty $R_{B}$. When the feature points reach the goal region, the agent achieves a reward of $R_{A}$. Based on these settings, the reward function $R_{t}$ is expressed as follows:

$$R_{t}=\sum_{j}^{N_{f}}1_{A}^{j}*R_{A}+\left(1-1_{A}^{j}\right)*\left(\left(1-1_{B}^{j}\right)*R_{j}^{\prime }+1_{B}^{j}*R_{B}\right)$$ (10)

where the definition of $1_{A}$ is same as that in (Eq. 7), and when the tissue significantly deforms during manipulation and leaves the surgical space, $1_{B}=1$; when the manipulation process is within the surgical space, $1_{B}=0$; $N_{f}$ is the total number of selected feature points; and $R_{j}^{\prime }=-\rho ∥d\left(t\right)∥$. If the target deformation is described by multiple points, such as curve-based deformation, we set the penalty signal $R^{\prime }$ equal to $-\rho \text{max}*∥d\left(t\right)∥$, which is not reduced by one.

Based on the aforementioned process, the agent can obtain the current state representation by interacting with the environment. Adjusting this factor coordinates the weightage between the IM mode mimicking the surgeon and the IM mode based on reinforcement learning to generate the current action using the ID-SAC algorithm. Additionally, with expert knowledge in designing the reward function, the agent acquires the cognitive expertise of experts, enhancing the intraoperative autonomous decision-making capabilities of the robot to accomplish manipulation tasks more effectively, thereby elevating the quality of surgical procedures. The training details are presented in Algorithm 2.

## Results

### Experimental setup

**Fig. 3.**
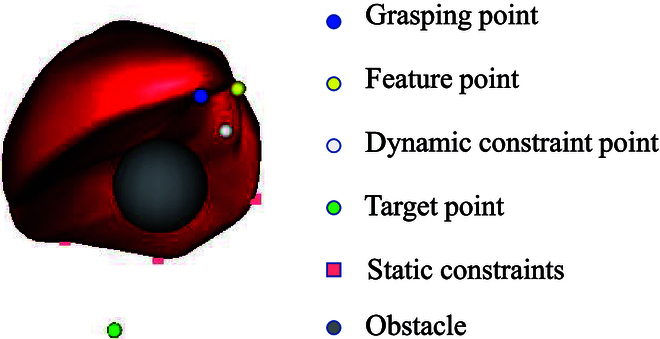
Simulation model of the human liver using SOFA.

Experiments are conducted based on a simulation model of the human liver on the simulation open framework architecture (SOFA) [25]. SOFA allows for creating three-dimensional models of the liver using medical imaging data such as computed tomography or magnetic resonance imaging. The simulated liver can closely match the size, shape, and structure of a real human liver, providing a realistic context for simulations. The framework can simulate the biomechanical behavior of liver tissue, including its elasticity, viscosity, and response to external forces. In SOFA simulations, the interaction between surgical tools and the liver model can be realistically rendered. This includes cutting, suturing, and retraction, which are common in liver surgeries. Force feedback and tool–tissue interactions aim to mimic real-life scenarios. Different simulation scenarios were designed for the three soft tissue target deformation tasks proposed in this study (Fig. 3). The liver models in the three task scenes were fixed in the simulation space using four known static constraints to prevent the liver from falling.

The static constraints are represented by pink squares: the blue points represent the grasping points, the yellow points represent the feature points, the green points represent the target points, and the white points represent unknown dynamic constraint points in the space. In the position-based deformation task, with one feature point and one target point, the target point is located far from the liver feature point. Additional obstacles are set on the manipulation trajectory from the feature point to the target point to further increase the manipulation task difficulty. The obstacles are represented by gray spheres with a radius of 0.4 in Fig. 3. In the curve-based deformation task, the feature points are a collection of four discrete points on a curve, and the target point corresponds to the feature points individually. In the experimental process, the four feature points must be manipulated to the corresponding target point simultaneously before the manipulation task is judged to be completed. In the region-based deformation task, the feature points are a collection of discrete points in the soft tissue of the region to be manipulated, with the region containing 13 feature points. All feature points need to enter the spherical target region outside the liver before the manipulation task is judged to have been achieved, and at this time, the yellow feature points will turn green.

Furthermore, obstacles and unknown dynamic interference scenes were added to the manipulation process for the position-based deformation task and for the curve- and the region-based deformation tasks, respectively, to further increase the experimental difficulty. The four manipulation scenes are defined as follows:

• NOND: Manipulation without obstacles and unknown dynamic disturbances;
• NOWD: Manipulation without obstacles but with unknown dynamic disturbances;
• WOND: Manipulation with obstacles but without unknown dynamic disturbances;
• WOWD: Manipulation with obstacles and unknown dynamic disturbances.

All networks comprise two hidden layers containing 256 units and are trained based on the Relu activation function using the Adam optimizer with a learning rate of 0.001, a discount factor γ of 0.99, and a batch size of 64. In this paper, every 1,000 steps were defined as one episode, and every 10,000 acts were recorded as an epoch. The training speed was defined as the epoch of the first completion of the training task, and the completion time was the time required to realize the task intelligence at a time. All experiments were performed using a single NVIDIA Titan RTX GPU with a 64-core CPU.

### Evaluation matrix

Based on four distinct intraoperative soft tissue manipulation tasks, this study validated the efficiency of the algorithm by assessing the agent’s ability to avoid obstacles during surgery and manipulate feature points to their target positions. When examining the algorithm for autonomous robotic soft tissue manipulation regarding the selection and assessment of grasp points, the trajectory rewards obtained from these points and the grasping success rate are defined as metrics to evaluate the quality of the selected grasp points. This study used the agent’s average reward and action value during training for intraoperative soft tissue manipulation tasks as metrics to evaluate the algorithm. Higher average rewards and action values indicate better strategies the agent adopts, whereas stability in these metrics signifies greater algorithm robustness and consistency.

Training speed is the time during which the agent completes the operational task for the first time during training. Task completion time is defined as the number of manipulation attempts the agent requires to accomplish an operational task using this algorithm. This study adopted training speed and task completion time as additional metrics to assess the algorithm. A faster training speed and shorter task completion time indicate that the algorithm requires less training time and achieves faster manipulation. Within error ranges of 0.2, 0.4, and 0.6, 100 random target points are generated separately. This study defined generalization as the probability of the agent completing these 100 randomly generated target manipulation tasks. This serves as a metric to validate the generalization capability of the algorithm.

Finally, evaluating the manipulation trajectory quality for soft tissue manipulation tasks is equally important. This study introduced nine commonly used metrics, including task manipulation time, total task manipulation distance, average operating speed along the *x*, *y*, and *z* axes, average manipulation acceleration along the *x*, *y*, and *z* axes, and trajectory straightness to assess the quality of the manipulation trajectory [26–30]. Because the trajectory is composed of discrete points $\left({\mathrm{Px}}_{i},\;{\mathrm{Py}}_{i}\right)$, the speed, acceleration, and trajectory straightness of one point are given as follows:

$$f^{\prime }\approx \frac{{\mathrm{Py}}_{i+1}-{\mathrm{Py}}_{i-1}}{{\mathrm{Px}}_{i+1}-{\mathrm{Px}}_{i-1}}$$ (11)

$$f^{\prime \prime }\approx \frac{{\mathrm{Py}}_{i+1}-2{\mathrm{Py}}_{i}+{\mathrm{Py}}_{i-1}}{\left({\mathrm{Px}}_{i+1}-{\mathrm{Px}}_{i}\right)\left({\mathrm{Px}}_{i}-{\mathrm{Px}}_{i-1}\right)}$$ (12)

$$k=\frac{|f^{\prime \prime }|}{{\left(1+{\left(f^{\prime }\right)}^{2}\right)}^{3/2}}$$ (13)

where ${\mathrm{Px}}_{i}$ and ${\mathrm{Py}}_{i}$ are three-dimensional vectors, and *N* is the number of discrete points in the trajectory.

A quality assessment index system for soft tissue manipulation trajectories was established and compared against trajectories manipulated independently by humans.

### Grasping point selection and assessment

**Fig. 4.**
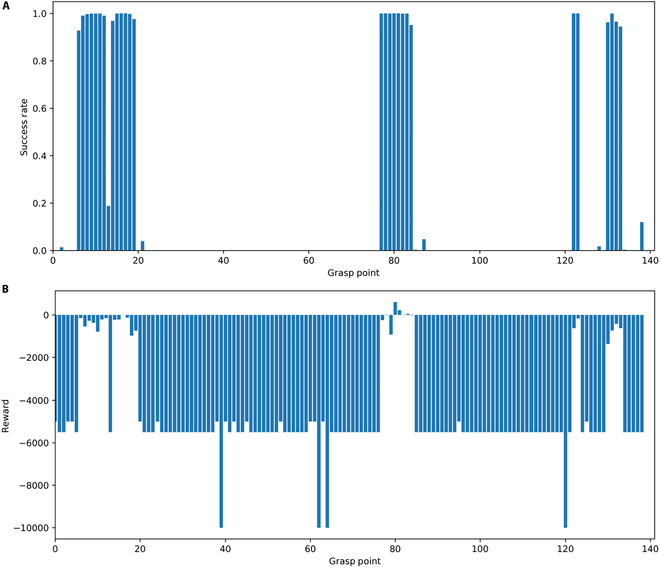
(A) Probability of successful manipulation in the human liver. (B) Evaluation results for each grasping point selected by the agent at that point.

The proposed soft tissue manipulation algorithm for grasp point selection and assessment was validated based on scenes of NOND and NOWD in intraoperative soft tissue manipulation tasks. Fig. 4A represents the success rates of completing the manipulation task for each graspable point on the liver model in the NOND task, as evaluated by the algorithm. The reward values obtained for each manipulation point in the liver model in the NOND task are presented in Fig. 4B. It is evident that if the success rate of the grasp point is high, the corresponding reward will be small. These results indicate that not all points in the liver model can accomplish the manipulation task. However, the proposed algorithm can identify grasp points with high reward values and success rates.

**Fig. 5.**
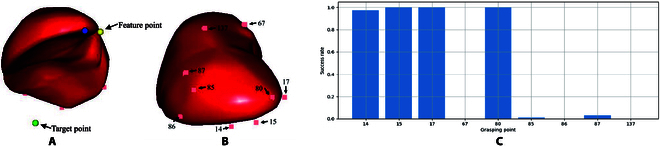
Distribution of grasp points in the simulation and the corresponding grasp success rate. (A) Feature point and its target point. (B) Distribution of grasp points. (C) Grasp success rate corresponding to the grasp points.

To demonstrate the grasp point selection and evaluation method more clearly, the distribution of some grasp points and their corresponding success rates are presented in Fig. 5. The target points were set at the anterior and inferior sides of the liver. The target point was inaccessible when manipulating the 137th grasp point. Similarly, the 67th grasp point was behind the liver, making the target point inaccessible. Moreover, although grasp points 85–87 demonstrated a certain success rate, they were not the optimal choice. Conversely, the 14th, 15th, and 17th grasp points were more consistent with the common sense of grasping and, therefore, exhibited a higher success rate.

**Fig. 6.**
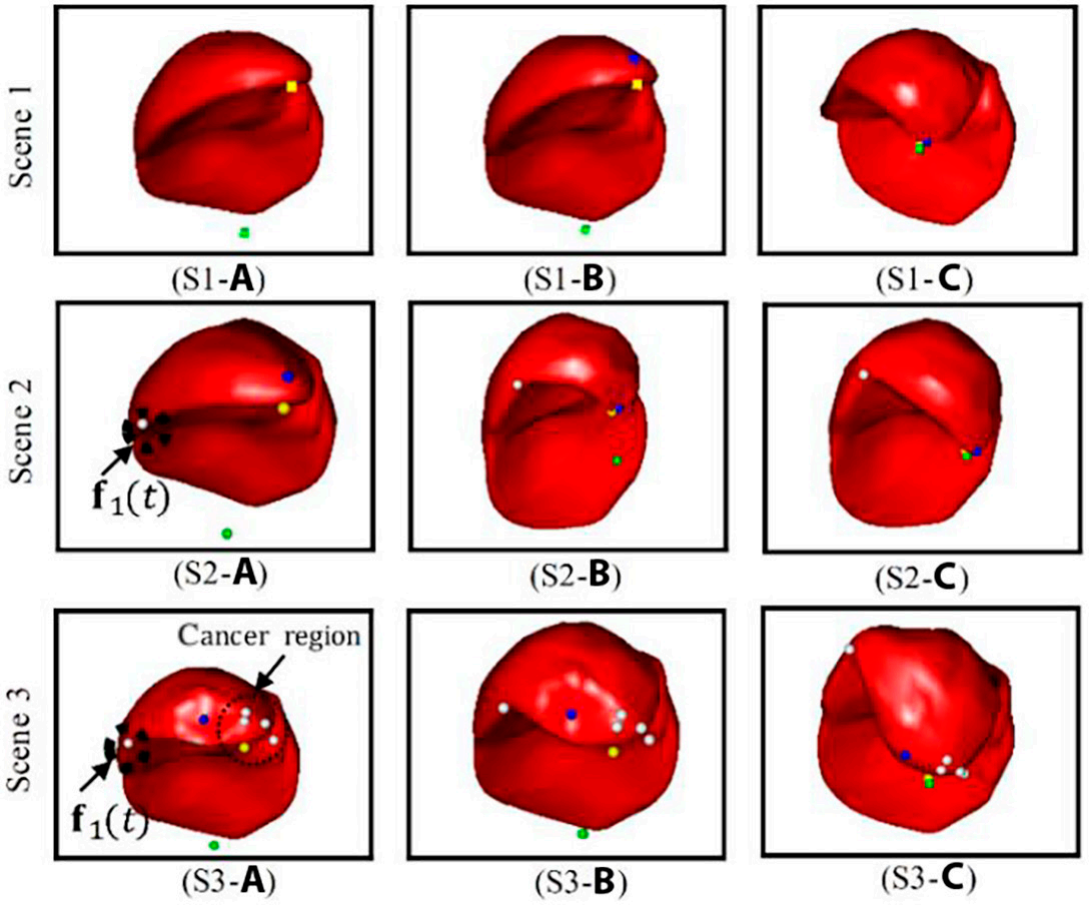
Successful example of autonomously generated initial grasp points. Scenes 1–3, respectively, represent three manipulation tasks: NOND, NOWD, and areas with lesions that cannot be grasped.

In the NOND task, the proposed autonomous soft tissue manipulation grasping point selection algorithm can select the optimal grasp point from all available points to execute the manipulation task, as illustrated in Fig. 6 (S1). The agent can select a suitable grasp point given a target deformation, denoted by the highest reward value, as depicted in Fig. 6 (S1-a). Moreover, in the NOWD task, the agent can select appropriate grasping points to accomplish the manipulation task, even when encountering unknown dynamic constraints affecting the liver tissue (Fig. 6 (S2)). Conversely, the experiments in this study further defined a region that could not be selected as the initial grasping point, similar to the tumor area (Fig. 6 (S3)). Even if the optimal grasp point cannot be selected within the tumor region, the agent can identify alternative points with high-reward values and success rates outside the tumor area to accomplish the manipulation task.

The experimental results confirmed the efficacy of the proposed autonomous grasping point selection and evaluation algorithm for soft tissue manipulation. This algorithm empowers the agent not only to select superior grasping points given a target deformation but also to assess the success rate of these grasping points, thereby further enhancing the autonomous decision-making capability of the robot in intraoperative soft tissue manipulation.

### Autonomous soft tissue manipulation

**Fig. 7.**
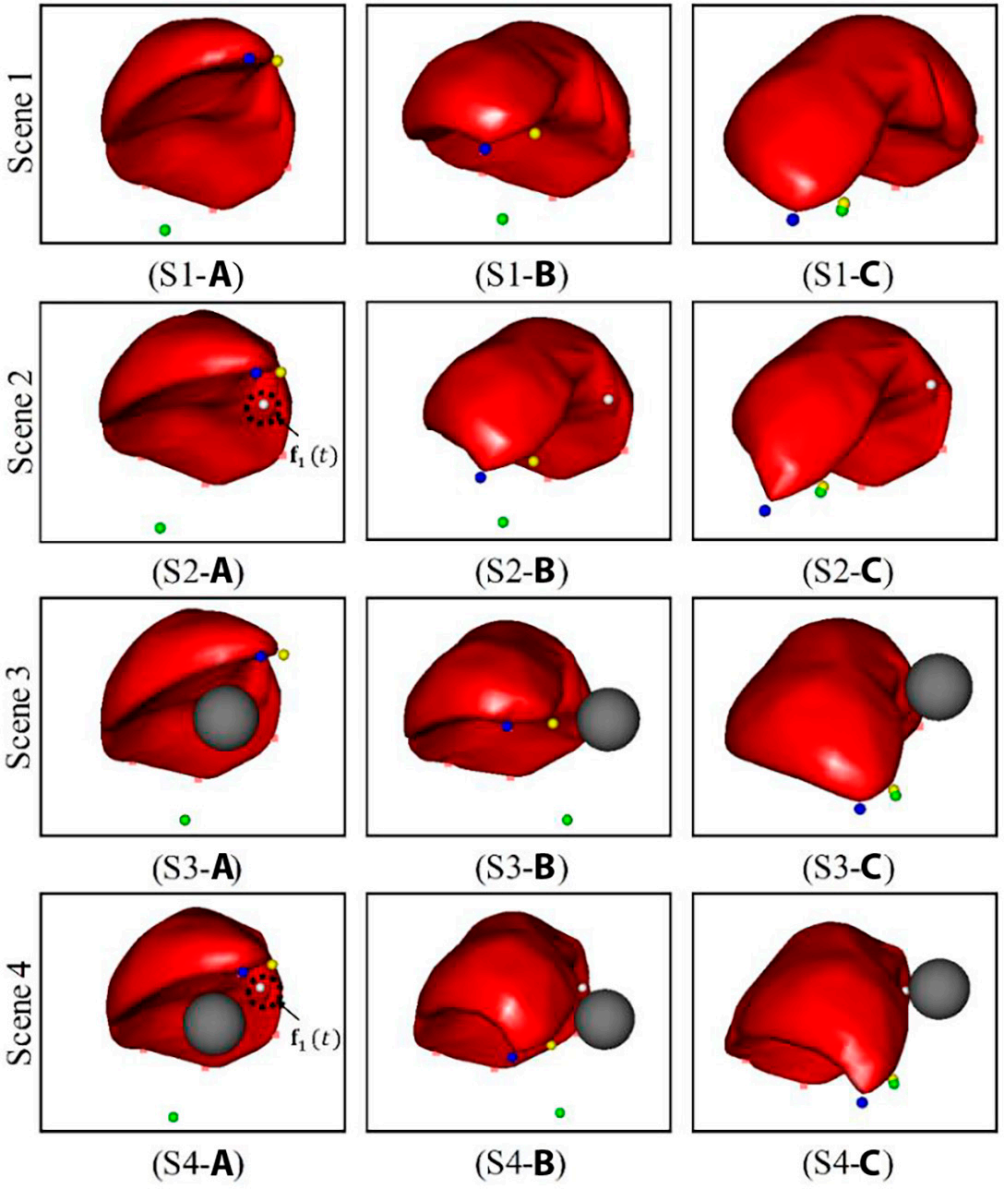
Successful examples of position-based soft tissue manipulation in four scenes. Scenes 1–4, respectively, represent four manipulation tasks: NOND, NOWD, WOND, and WOWD.

Based on the proposed autonomous grasping point selection and evaluation algorithm, the ID-SAC algorithm was trained using the grasping points selected for soft tissue manipulation tasks. Considering the four position-based autonomous soft tissue deformation manipulation tasks, the ID-SAC algorithm introduced in this study effectively accomplished autonomous soft tissue manipulation tasks (Fig. 7). Fig. 7 (S1 and S2) and Fig. 6 (S1 and S2) compare the results in the NOND and NOWD scenes, where different grasp points are used. Most importantly, the agent can even complete the manipulation task in the presence of obstacles (Fig. 7 (S3 and S4)). Fig. 7 (S3) illustrates that the agent can effectively avoid the obstacle (S3-b) and reach the target point (S3-c), even if the obstacle is located directly under the liver. Therefore, in the case of unknown dynamic constraints, it remains possible to successfully avoid obstacles (S4-b) and reach the target point (S4-c). The influence of the unknown constraints on the manipulation is evident in the comparison of Fig. S3-c and S4-c. The ID-SAC algorithm exhibited the ability to navigate around obstacles when faced with unknown dynamic disturbances.

**Fig. 8.**
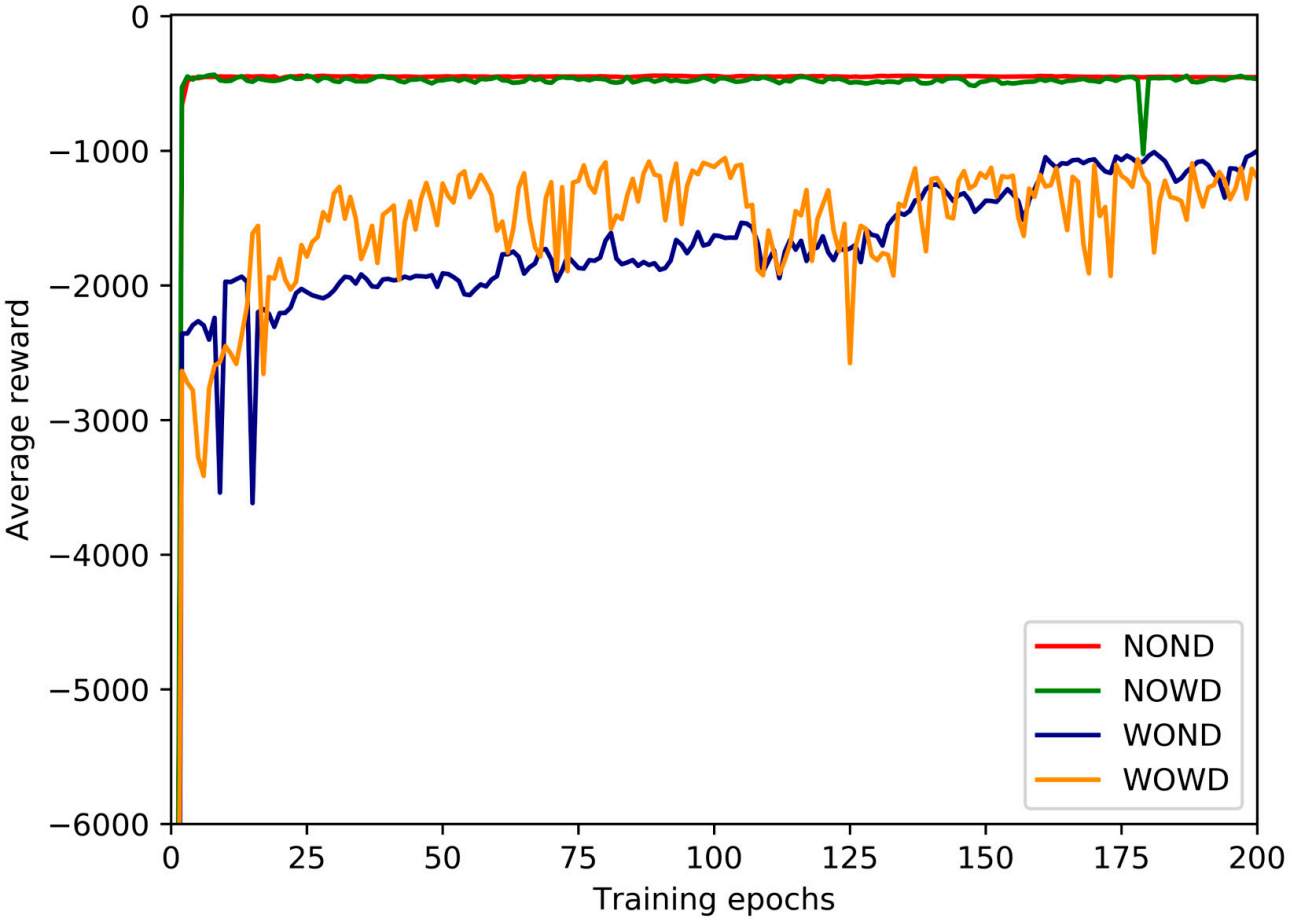
Average reward curves for agent training in four scenes based on position-based deformation.

Fig. 8 illustrates the average reward for each training cycle during the initial 200 cycles. As training progressed, the average rewards for all tasks gradually stabilized, indicating the convergence of the algorithm across the four task scenes. The introduction of unknown dynamic disturbances increases task complexity. However, the unpredictable motion of the dynamic interference points indirectly encourages exploration by the agent, thereby facilitating better task achievement. Initially, the action value of the states with obstacles and disturbances was lower than those with obstacles but no disturbances. Nonetheless, the action value of states with obstacles and disturbances steadily increased for training owing to the increased exploration.

**Fig. 9.**
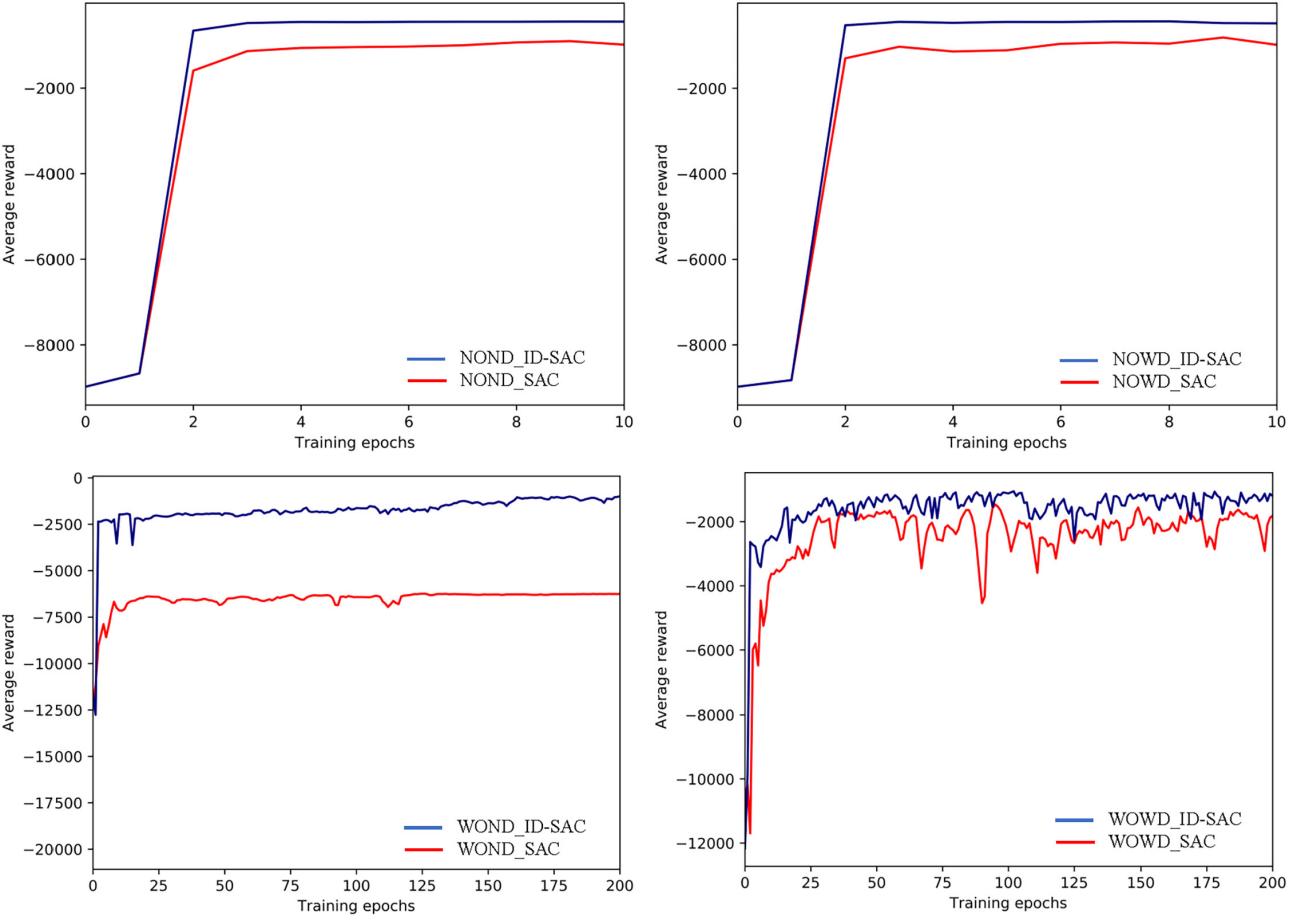
Average reward curves for the ID-SAC and SAC algorithms for four scenes based on position-based deformation.

The comparative results of the ID-SAC and SAC algorithms are depicted in Fig. 9. As the training progressed, the ID-SAC algorithm demonstrated superior average rewards over the SAC algorithm across the four task scenes based on location. Despite the capability of the SAC algorithm to enhance agent exploration in long-distance scenes of intraoperative soft tissue manipulation tasks, its efficacy diminishes in high-dimensional and distant manipulation tasks. In such scenarios, the SAC algorithm fails to adequately explore the vicinity of the target, resulting in an inability to perform manipulations across the four tasks and restricting the agent’s manipulation behavior within proximity to the initial position.

**Fig. 10.**
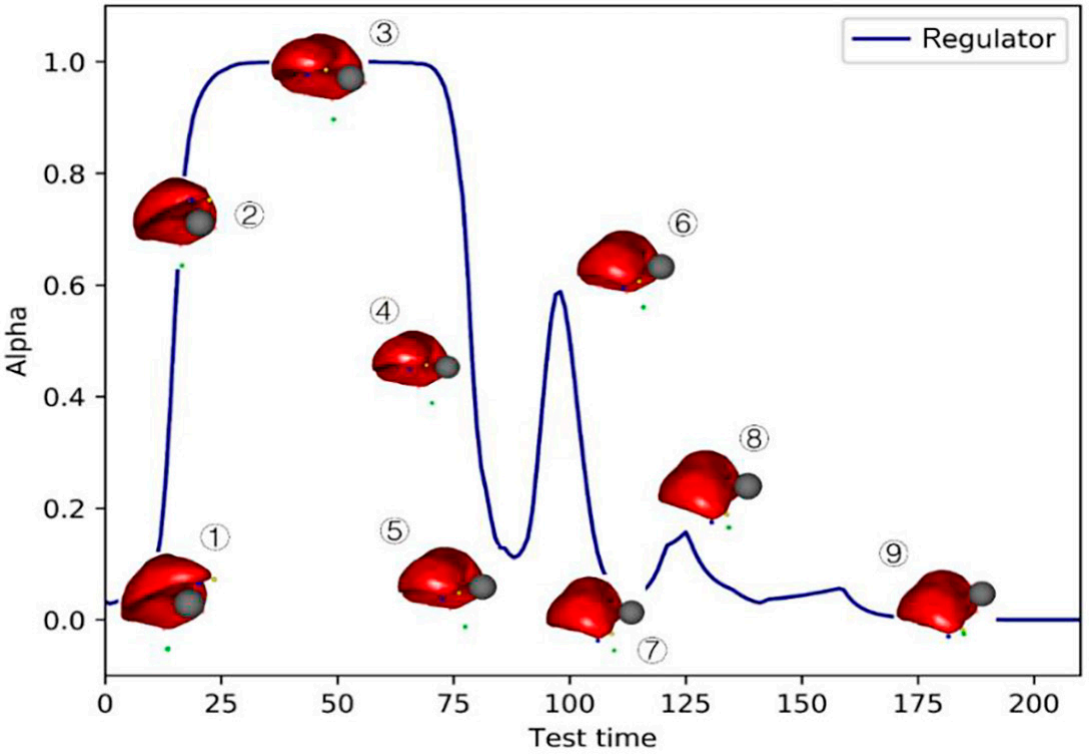
Regulatory factor change curves for two modes of soft tissue manipulation.

The core principle of the ID-SAC algorithm is to autonomously combine the learned regulatory factors of simulated IM patterns and reinforcement-learning-based deliberate manipulation patterns, thereby improving the autonomous decision-making capability of the robot to achieve the desired manipulations. Consequently, this study further validated whether the proposed fusion strategy can enhance the autonomous decision-making ability of the robot. The variation curve of the autonomously generated regulatory factors during the completion of the soft tissue manipulation process in the WOND soft tissue manipulation task scene is depicted in Fig. 10. Subfigures 1–9 in Fig. 10 represent the soft tissue state during the manipulation process, demonstrating the successful completion of the manipulation task. Subfigure 1 represents the initial phase of the manipulation task, where the soft tissue was at a distance from the obstacle. The agent initiated the soft tissue manipulation toward the target point, following a simulated IM mode akin to the doctor’s approach. The tissue progressively approaches the obstacle as it lies along the trajectory between the feature and target points during the simulated IM. Subsequently, the agent activates a deliberate manipulation mode based on reinforcement learning, transitioning the soft tissue, as depicted in Subfigure 2. The agent autonomously maneuvers the tissue to navigate around the obstacle, as evident in Subfigure 3. The agent maintains the deliberate manipulation mode based on reinforcement learning until the soft tissue has preliminarily avoided the obstacle. Transitioning from Subfigure 4 to 5, the agent discerns an obstacle-free trajectory while manipulating the tissue toward the target deformation.

Consequently, the agent reduces the weightage of the deliberate manipulation mode based on reinforcement learning and increases the ratio of the simulated intuitive manipulation mode (Subfigure 6). Uncertain deformations may occur during tissue manipulation (Subfigure 6), where a portion of the lateral soft tissue of the liver approaches the obstacle. The agent reactivates the deliberate manipulation mode based on reinforcement learning until the tissue segment circumvents the obstacle. To ensure swift manipulation, the agent resumes the simulated IM mode (Subfigure 7). The backside tissue of the liver approaches the obstacle, compelling the agent to re-engage the deliberate manipulation mode based on reinforcement learning for meticulous manipulation until complete avoidance is achieved (Subfigure 8). The agent continues to alternate between modes, swiftly manipulating soft tissue to achieve target manipulation.

The aforementioned process extensively validates the ability of the agent to swiftly adapt its manipulation mode according to the current soft tissue manipulation scene, generating optimal manipulation strategies to accomplish the manipulation task. The manipulation strategies derived from this algorithm demonstrate a thought process akin to human cognition. The ID-SAC algorithm, derived from the fusion strategy of soft tissue manipulation based on deep reinforcement learning, empowers robots with decision-making capabilities that resemble those of humans. This effectively enhances the intraoperative decision-making process of the robot, enabling it to better execute soft tissue manipulation tasks and improve the quality of surgeries.

Table 1 lists the training speed, task completion time, and generalization test results of the ID-SAC algorithm across the four task scenes. Compared to the SAC algorithm, the algorithm proposed in this study demonstrated faster training speeds and shorter task completion times and exhibited certain levels of generalization while accomplishing manipulation tasks. The ID-SAC algorithm, designed to emulate a simulated IM mode akin to a medical professional’s approach, improves the agent’s ability to explore distant regions, thereby addressing the issue of insufficient exploration capabilities in distant scenarios. This enhanced level of exploration further assists in optimizing fine manipulation modes based on reinforcement learning. The synergistic interplay between the regulator factor and exploration mechanism mutually enhances the performance of the algorithm, significantly improving the realization of intraoperative soft tissue manipulation tasks within complex scenes.

**Table 1.** Results of agent manipulation based on the ID-SAC algorithm in four tasks based on position-based deformation

Scene	Training speed	Task completion time	Generalizability		
			$v_{d}\pm 0.2$	$v_{d}\pm 0.4$	$v_{d}\pm 0.6$
NOND	1	174	100%	100%	97%
NOWD	1	196	100%	100%	91%
WOND	12	277	100%	96%	81%
WOWD	55	273	69%	55%	54%

**Fig. 11.**
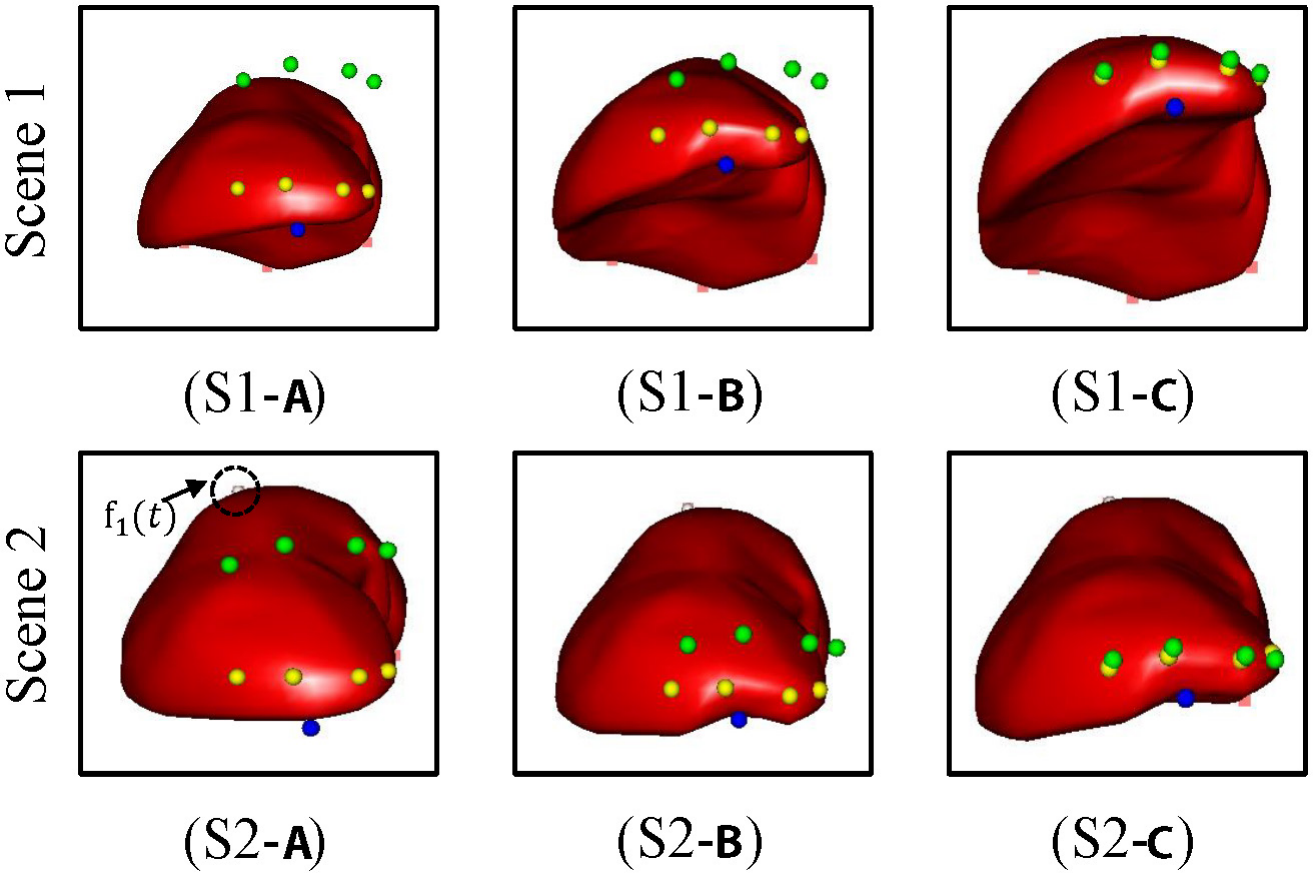
Successful examples of curve-based soft tissue manipulation in two scenarios. Scenes 1 and 2, respectively, represent two manipulation tasks: NOND and NOWD.

**Fig. 12.**
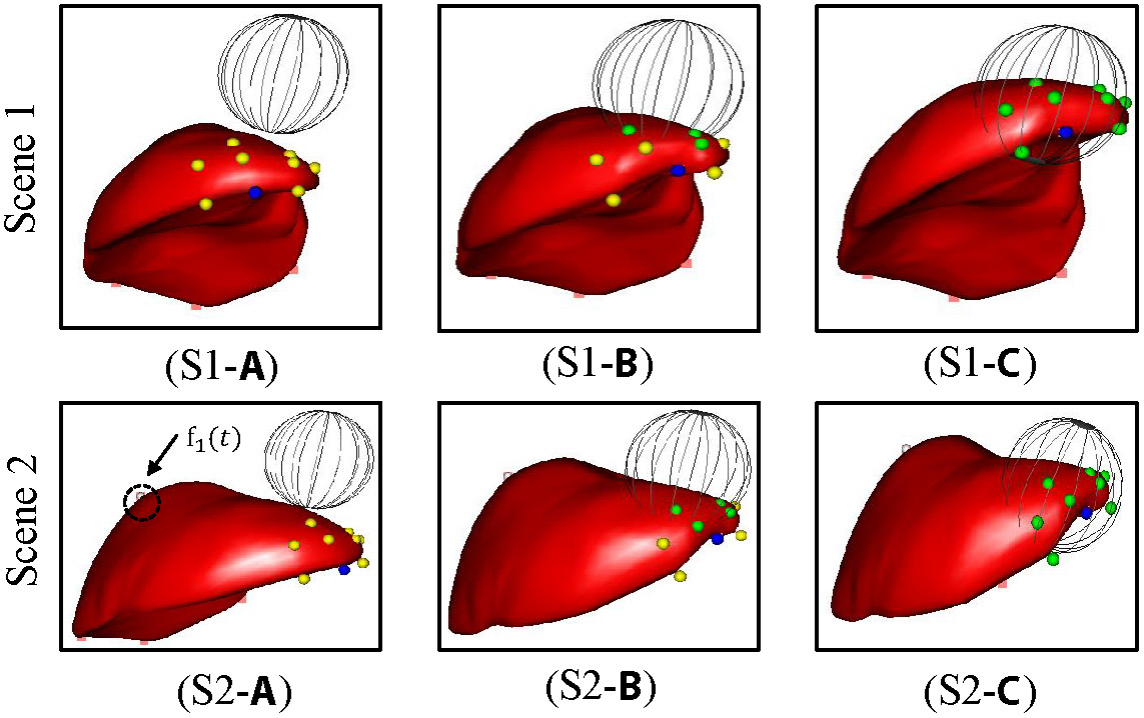
Successful examples of region-based soft tissue manipulation in two scenarios. Scenes 1 and 2, respectively, represent two manipulation tasks: NOND and NOWD.

To further validate the effectiveness of the algorithm in extensive manipulation scenes, we conducted assessments of deformation manipulation tasks based on the curves and regions within the NOND and NOWD tasks (Fig. 11 and 12). The results indicated that the ID-SAC algorithm adeptly manipulates flexible bodies to accurately reach target curves and regions. This algorithm can be used to manipulate multiple target points across various tasks.

### Compared with human manipulation trajectories

This study used the ID-SAC algorithm to generate autonomous manipulation trajectories of agents in position-based intraoperative soft tissue manipulation tasks. These trajectories were then compared with human-manipulated trajectories based on the defined criteria of the soft-tissue manipulation trajectory quality evaluation index system. The results indicated that the agents outperformed humans in task completion time and total distance traveled across all four tasks (Tables 2 and 3). However, in these scenarios, the agents exhibited a faster average manipulation speed and acceleration than humans.

**Table 2.** Comparison of the manipulation trajectories from the ID-SAC and the human

Scene	Manipulation subject	Task time	Total distance	Average speed		
				*x*	*y*	*z*
NOND	Agent	162	3.83	0.04	−1.03	0.49
	Human	544	6.04	0.03	−0.32	0.14
NOWD	Agent	179	4.29	0.09	−1.00	0.48
	Human	731	5.97	0.04	−0.25	0.12
WOND	Agent	216	6.48	0.02	−0.78	0.38
	Human	872	6.86	0.02	−0.20	0.10
WOWD	Agent	245	7.19	0.07	−0.73	0.36
	Human	982	13.0	0.01	−0.20	0.09

**Table 3.** Comparison of the manipulation trajectories from the ID-SAC and the human (continue)

Scene	Manipulation subject	Trajectory straightness	Average acceleration		
			*x*	*y*	*z*
NOND	Agent	0.01	−0.15	0.41	−0.46
	Human	0.05	−0.02	−0.02	0.03
NOWD	Agent	0.02	−0.40	0.38	−0.42
	Human	0.05	−0.02	−0.003	0.003
WOND	Agent	0.04	−0.09	0.32	0.32
	Human	0.03	−0.01	−0.001	−0.01
WOWD	Agent	0.05	−0.07	0.28	−0.29
	Human	13.04	0.01	−0.20	0.09

When comparing the trajectories of agents and humans in tasks involving NOND and NOWD, the trajectories generated by agents displayed superior trajectory smoothness compared to those generated by humans. Agents managed to execute tasks more smoothly. The agent used deliberate manipulation actions to avoid obstacles in proximity in tasks involving WOND and WOWD, owing to the agent’s capacity to perform actions between IM and deliberate manipulation modes based on reinforcement learning. In other instances, it swiftly executed manipulation tasks using the IM mode. In contrast to humans, who initially opted to perform tasks at a distance, the actions performed by agents caused relatively curved trajectories, resulting in slightly higher trajectory smoothness. This approach led to shorter task completion times and less overall distance while maintaining trajectory smoothness comparable to that of humans.

Comparing the trajectory quality between robot autonomous decisions and human-independent manipulation revealed that the robot’s autonomous decisions require a shorter manipulation time and total distance, resulting in smoother trajectories. Furthermore, they exhibit faster manipulation speeds and accelerations. The robot can assimilate the expertise of skilled practitioners, acquire advanced cognitive knowledge, and formulate superior manipulation strategies. This enhancement in autonomous manipulation capabilities enables the more efficient completion of surgical manipulation tasks, thereby improving surgical quality.

## Conclusion

To enhance surgical quality, this study introduced a human-machine collaborative decision-making approach in which the robot assumed the decision-making role. It proposed an algorithm using a deep Q-network for autonomous robot-assisted soft tissue manipulation, explicitly focusing on learning initial grasp point selection strategies. This algorithm effectively identifies the grasp points conducive to executing manipulation tasks, thereby enhancing the autonomy of the robot during surgical procedures. Simultaneously, this study presented a fusion strategy for soft tissue manipulation based on deep reinforcement learning to address intraoperative soft tissue manipulation and obstacle avoidance challenges. By incorporating a regulatory factor, this strategy autonomously integrates multiple manipulation modes. The resultant ID-SAC algorithm significantly enhances the robot’s autonomous decision-making abilities, enabling it to execute soft tissue manipulation and obstacle avoidance tasks effectively. The trajectory quality achieved through autonomous decision-making surpasses that of individual medical practitioners, leading to an improved quality of surgical manipulations.

## Data Availability

Data are available upon reasonable request.

## References

[1] Dupont PE, Nelson BJ, Goldfarb M, Hannaford B, Menciassi A, O’Malley MK, Simaan N, Valdastri P, Yang GZ. A decade retrospective of medical robotics research from 2010 to 2020. Sci Robot. 2021;6(60):eabi8017. 34757801 10.1126/scirobotics.abi8017 PMC8890492

[2] Shadman A, Decker RS, Opfermann JD, Leonard S, Krieger A, Kim PCW. Supervised autonomous robotic soft tissue surgery. Sci Transl Med. 2016;8(337):337ra64. 10.1126/scitranslmed.aad9398 27147588

[3] Saeidi H, Opfermann J, Kam M, Wei S, Leonard S, Hsieh MH, Kang JU, Krieger A. Autonomous robotic laparoscopic surgery for intestinal anastomosis. Science. Sci Robot. 2022;7(62):eabj2908. 35080901 10.1126/scirobotics.abj2908 PMC8992572

[4] Nguyen T, Nguyen ND, Bello F, Nahavandi S. A new tensioning method using deep reinforcement learning for surgical pattern cutting. IEEE Int Conf Industrial Technol. 2019;2019:1339–1344.

[5] Navarro-Alarcon D, Liu YH, Romero JG, Li P. Model-free visually servoed deformation control of elastic objects by robot manipulators. IEEE Trans Robot. 2013;29:1457–1468.

[6] Shahkoo AA, Abin AA. Autonomous tissue manipulation via surgical robot using deep reinforcement learning and evolutionary algorithm. IEEE Trans Med Robot Bionics. 2023;5(1):30–41.

[7] Mo H, Ouyang B, Xing L, Dong D, Liu Y, Sun D. Automated 3-d deformation of a soft object using a continuum robot. IEEE Trans Autom Sci Eng. 2020;18(4):2076–2086.

[8] Ouyang B, Mo H, Chen H, Liu Y, Sun D. Robust model-predictive deformation control of a soft object by using a flexible continuum robot. IEEE/RSJ Inter Conf Intell Robots Syst. 2018;2018:613–618.

[9] Essahbi N, Bouzgarrou BC, Gogu G. Soft material modeling for robotic manipulation. Appl Mech Mater. 2012;162:184–193.

[10] Kaufmann P, Martin S, Botsch M, Gross M. Flexible simulation of deformable models using discontinuous Galerkin FEM. Graph Model. 2009;71(4):153–167.

[11] Pan J, Bai J, Zhao X, Hao A, Qin H. Real-time haptic manipulation and cutting of hybrid soft tissue models by extended position-based dynamic. Comput Animat Virt W. 2015;26(3–4):321–335.

[12] Navarro-Alarcon D, Yip HM, Wang Z, Liu YH, Zhong F, Zhang T, Li P. Automatic 3-d manipulation of soft objects by robotic arms with an adaptive deformation model. IEEE Trans Robot. 2016;32(2):429–441.

[13] Hu Z, Han T, Sun P, Pan J, Manocha D. 3-d deformable object manipulation using deep neural networks. IEEE Robot Autom Lett. 2019;4(4):4255–4261.

[14] Akkaya I, Andrychowicz M, Chociej M, Litwin M, Grew BM, Petron A, Paino A, Plappert M, Powell G, Ribas R, et al. Solving Rubik’s cube with a robot hand. arXiv. 2019. 10.48550/arXiv.1910.07113

[15] Pedram SA, Ferguson PW, Shin C, Mehta A, Dutson EP, Alambeigi F, Rosen J. Toward synergic learning for autonomous manipulation of deformable tissues via surgical robots: An approximate q-Learning approach. Paper presented at: IEEE RAS/EMBS International Conference for Biomedical Robotics and Biomechatronics. 29 Nov–01 Dec 2020; New York, NY, USA. p. 878–884.

[16] Fujimoto S, Meger D, Precup D. Off-policy deep reinforcement learning without exploration. Proc Mach Learn Res. 2019;97:2052–2062.

[17] Jing M, Ma X, Huang W, Sun F, Yang C, Fang B, Liu H. Reinforcement learning from imperfect demonstrations under soft expert guidance. Proc AAAI Conf Artific Intell. 2020;34:5109–5116.

[18] Viswanath V, Grannen J, Sundaresan P, Thananjeyan B, Balakrishna A. Disentangling dense multi-cable knots. IEEE/RSJ Inter Conf Intell Robots Syst. 2021;2021:3731–3738.

[19] Huang J, Cai Y, Chu X, Taylor RH, Au KWS. Non-fixed contact manipulation control framework for deformable objects with active contact adjustment. IEEE Robot Autom Lett. 2021;6(2):2878–2885.

[20] Haarnoja T, Zhou A, Abbeel P, Levine S. Soft actor-critic: Off-policy maximum entropy deep reinforcement learning with a stochastic actor. Proc Mach Learn Res. 2018;80:1861–1870.

[21] Haarnoja T, Zhou A, Hartikainen K, Tucker G, Ha S, Tan J. Soft actor-critic algorithms and applications. arXiv. 2018. 10.48550/arXiv.1812.05905

[22] Sutton RS, Barto AG. *Reinforcement learning, second edition: An introduction (2nd ed.)*. Cambridge (MA): The MIT Press; 2018.

[23] Mnih V, Kavukcuoglu K, Silver D, Rusu AA, Veness J, Bellemare MG, Graves A, Riedmiller M, Fidjeland AK, Ostrovski G, et al. Human-level control through deep reinforcement learning. Nature. 2015;518:529–533. 25719670 10.1038/nature14236

[24] Rusu RB, Blodow N, Beetz M. Fast point feature histograms (FPFH) for 3D registration. IEEE Int Conf Robot Autom. 2009;2019:1848–1853.

[25] Faure F, Duriez C, Delingette H, Allard J, Gilles B, Marchesseau S, Talbot H, Courtecuisse H, Bousquet G, Peterlik I, et al. SOFA: A multi-model framework for interactive physical simulation. In: Payan Y, editor. *Soft tissue biomechanical modeling for computer assisted surgery*. Berlin, Heidelberg: Springer; 2012. p. 283–321.

[26] Ergina PL, Cook JA, Blazeby JM, Boutron I, Clavien PA, Reeves BC, Seiler CM, Balliol Collaboration, Altman DG, Aronson JK, et al. Challenges in evaluating surgical innovation. Lancet. 2009;374(9695):1097–1104. 19782875 10.1016/S0140-6736(09)61086-2 PMC2855679

[27] Judkins TN, Oleynikov D, Stergiou N. Objective evaluation of expert and novice performance during robotic surgical training tasks. Surg Endosc. 2009;23(23):590–597. 18443870 10.1007/s00464-008-9933-9

[28] Nisky I, Okamura AM, Hsieh MH. Effects of robotic manipulators on movements of novices and surgeons. 612. Surg Endosc. 2014;28(7):2145–2158. 24519031 10.1007/s00464-014-3446-5 PMC8101070

[29] Goh A, Goldfarb D, Sander J, Miles B, Dunkin B. Global evaluative assessment of robotic skills: Validation of a clinical assessment tool to measure robotic surgical skills. J Urol. 2012;187(1):247–252. 22099993 10.1016/j.juro.2011.09.032

[30] Darzi A, Mackay S. Skills assessment of surgeons. Surgery. 2002;131(2):121–124. 11854687 10.1067/msy.2002.115831

